# Promiscuous Chemokine Antagonist (BKT130) Suppresses Laser-Induced Choroidal Neovascularization by Inhibition of Monocyte Recruitment

**DOI:** 10.1155/2019/8535273

**Published:** 2019-08-05

**Authors:** Shira Hagbi-Levi, Michal Abraham, Liran Tiosano, Batya Rinsky, Michelle Grunin, Orly Eizenberg, Amnon Peled, Itay Chowers

**Affiliations:** ^1^Department of Ophthalmology, Hadassah-Hebrew University Medical Center, Jerusalem 91120, P.O.B 12000, Israel; ^2^Biokine Therapeutics Ltd., Science Park, Ness Ziona, Israel; ^3^Goldyne Savad Institute of Gene Therapy, Hadassah-Hebrew University Medical Center, Jerusalem 91120, P.O.B 12000, Israel

## Abstract

**Background:**

Age-related macular degeneration (AMD), the most common cause of blindness in the developed world, usually affects individuals older than 60 years of age. The majority of visual loss in this disease is attributable to the development of choroidal neovascularization (CNV). Mononuclear phagocytes, including monocytes and their tissue descendants, macrophages, have long been implicated in the pathogenesis of neovascular AMD (nvAMD). Current therapies for nvAMD are based on targeting vascular endothelial growth factor (VEGF). This study is aimed at assessing if perturbation of chemokine signaling and mononuclear cell recruitment may serve as novel complementary therapeutic targets for nvAMD.

**Methods:**

A promiscuous chemokine antagonist (BKT130), aflibercept treatment, or combined BKT130+aflibercept treatment was tested in an *in vivo* laser-induced model of choroidal neovascularization (LI-CNV) and in an ex vivo choroidal sprouting assay (CSA). Quantification of CD11b+ cell in the CNV area was performed, and mRNA levels of genes implicated in CNV growth were measured in the retina and RPE-choroid.

**Results:**

BKT130 reduced the CNV area and recruitment of CD11b+ cells by 30-35%. No effect of BKT130 on macrophages' proangiogenic phenotype was demonstrated ex vivo, but a lower *VEGFA* and *CCR2* expression was found in the RPE-choroid and a lower expression of *TNFα* and *NOS1* was found in both RPE-choroid and retinal tissues in the LI-CNV model under treatment with BKT130.

**Conclusions:**

Targeting monocyte recruitment via perturbation of chemokine signaling can reduce the size of experimental CNV and should be evaluated as a potential novel therapeutic modality for nvAMD.

## 1. Introduction

Dysregulation of the complement and systemic immune systems has been associated with the pathogenesis of age-related macular degeneration (AMD). Genetic, histological, and biochemical studies have associated the alternative complement pathway with the disease [1–8]. Lymphocytes, mononuclear cells, and particularly monocytes and macrophages were also implicated in AMD [3, 9–23]. In fact, infiltration of monocytes to the retina was found to be essential for the development of choroidal neovascularization (CNV) [18, 24]. Increased numbers of CD56+ T cells have been detected in the blood of AMD patients when compared to age-matched controls [25], and the interaction of T cells and M1 macrophages was reported during the stages of AMD [23]. Once recruited to the eye, monocytes differentiate to macrophages that can exert a proangiogenic effect in the context of neovascular AMD (nvAMD), an effect that may be exacerbated in aging [17, 18, 22, 26–31]. Activated macrophages from nvAMD patients might exert a more significant proangiogenic effect compared with macrophages from age-matched controls [22]. Several macrophage-derived cytokines, in addition to VEGF, can mediate CNV growth [32, 33]. Accordingly, perturbation of monocyte recruitment and/or function may potentially result in suppression of CNV growth that may potentially complement anti-VEGF-based therapies.

Chemokines and their receptors play a critical role in the progression of autoimmune and inflammatory diseases such as AMD. Multiple chemokines were found to be involved in the development of this pathology [34–38]. For example, a transcriptome-wide analysis of the AMD donor retinas suggested that CCL2, IP-10, MIG, and I-TAC are upregulated in all forms of the disease [39]. CXCR3 is one of the mammalian chemokine receptors, promoting chemotaxis and cell proliferation. This receptor binds to three major chemokine ligands: IP-10, MIG, and I-TAC. CXCR3 expression and IP-10 were elevated in the RPE-choroid fractions of the laser-induced CNV eyes compared with nontreated fellow eyes [40]. Our group has reported an increased expression of other chemokine receptors, namely, CCR1 and CCR2, in the CD14+CD16+ subset of monocytes from neovascular AMD (nvAMD) patients [41]. CCR2 is a major chemokine receptor that is also potentially involved in macrophage activation and recruitment in AMD [32]. In accordance with the high levels of MCP-1, the ligand for CCR2 was detected in the aqueous humor of patients with AMD [42, 43], and macrophages have been found in the vicinity of drusen areas of retinal pigment epithelium (RPE) atrophy, Bruch's membrane rupture, and choroidal neovascularization (CNV) in histological sections from AMD eyes [44–49].

Targeting a single chemokine, or its receptor, in an attempt to reduce macrophage recruitment to the retina was contemplated as a potential treatment for AMD. This approach is limited by the redundancy of the chemokine signaling system and by the nonexclusive nature of ligand-receptor interactions which characterizes it [50–52]. Here, we suggest an alternative approach involving antagonizing multiple chemokine signaling pathways simultaneously. Accordingly, a recent study demonstrated the efficiency of a broad-spectrum chemokine inhibitor (NR58-3.14.3) in modulating macrophage-mediated inflammation in light-induced retina injury [53].

BKT130 is a novel promiscuous chemokine-binding peptibody which has the ability to bind and inhibit multiple inflammatory chemokines, such as CCL2 (ligand for CCR2), CCL5 (binding CCR5), IP-10, MIG, and I-TAC (binding to CXCR3) [54]. This novel peptibody was already proven to have a therapeutic effect in autoimmune and inflammatory pathologies by inhibition of the recruitment of immune cells, inflammation, and disease progression in rodent models for rheumatoid arthritis (RA) and multiple sclerosis (MS) [54]. BKT130 was also found to inhibit melanoma and pancreatic tumor cell growth in mice [54]. In this study, we assessed the effect of this chemokine antagonist in a rodent model for laser injury-induced CNV and in complementary *in vitro* experiments.

## 2. Materials and Methods

### 2.1. Laser-Induced Model of CNV (LI-CNV) and Experimental Groups

LI-CNV was generated in adult Long-Evans rats (8-12 weeks old). Animals were treated in accordance with the guidelines of the Association for Research in Vision and Ophthalmology (ARVO). Experiments were conducted with the approval of the institutional animal care ethics committee. Before each procedure, rats were anesthetized by intraperitoneal injections of a mixture of 85% ketamine (Bedford Laboratories, Bedford, OH) and 15% xylazine (VMD, Arendonk, Belgium). Local anesthesia using oxybuprocaine HCL 0.4% (Localin) drops (Fisher Pharmaceuticals, Tel-Aviv, Israel) was applied to each eye 10 minutes before intravitreal injections or laser photocoagulation.

Laser burns (5-7 burns per eye) were generated as previously described [55]. Intravitreal injections were performed using a PLI-100 Pico-Injector (Medical System Corp., Greenvale, NY) as we have previously described [22]. Intravitreal injections of either 4 *μ*l of 5 mg/ml BKT130 (Biokine, Ness Ziona, Israel) (*n* = 9 eyes), 1 *μ*l of 40 mg/ml aflibercept (Bayer Pharma AG, Berlin, Germany) (*n* = 8), a combination of 4 *μ*l BKT130 and 1 *μ*l of aflibercept (*n* = 8), or 4 *μ*l of PBS solution (*n* = 10) were provided. BKT130 dosage was according to a previous study which tested dose response and kinetic analysis in vivo [54], and aflibercept dosage was according to that used in human eyes which was adjusted according to the size of the rat eye. All intravitreal injections were performed at the time of the laser burn injury and 5 days later. Antibiotic ointment (5% Synthomycine) was applied after the injection. RPE-choroid flat mounts were dissected and processed for isolectin staining 10 days following the laser injury as we have previously described [22]. The contralateral retina of the same rat was homogenized and frozen at -80 for RNA extraction using TRI-Reagent (Sigma-Aldrich, Munich, Germany).

### 2.2. CNV Quantification

RPE-choroid flat mounts were fixated for one hour in 4% PFA and suspended overnight in isolectin solution (GS-Ib4 Alexa 594 staining solution, Molecular Probes, Eugene, Oregon) containing 200 mM NaN_3_ and 1 mM CaCl_2_. Flat mounts were then washed 6 times for 20 minutes in PBS and embedded on a slide with a mounting medium. Isolectin images of the RPE-choroid flat mounts were viewed using a fluorescent microscope (Olympus BX41, Tokyo, Japan). Background was controlled by setting the exposure parameters as such so that they provided no detectable signal for the control nonimmune serum-stained rat flat mount. These same parameters were maintained while capturing all images from the test sections. Images were photographed with an Olympus DP70 digital camera.

The CNV area around each laser injury was measured using the ImageJ software [56]. The optic disc was removed to avoid autofluorescence from background counts. The scale was set to translate pixels into mm^2^, threshold was set on an unstained negative control, and these settings were used as background for all images. In order to calculate the average area of each CNV, we calculated the stained area of particles above the size of 60 pixels and divided it by the number of laser burns in the eye. The average CNV area of each eye was then calculated, and the mean CNV area of the four groups was compared.

### 2.3. Immunohistochemistry

Immunohistochemistry of the fixed retinas was performed for mononuclear phagocyte (CD11b+) cell count. In brief, mouse monoclonal anti-CD11b (Abcam, ab78457, 1 : 100) was used as the primary antibody for the rat mononuclear phagocytes. A donkey anti-mouse (Abcam, ab150110, 1 : 100) was used as the secondary antibody. Retinal flat mounts were first permeabilized and blocked for 3 hours at room temperature (RT) with blocking solution containing 0.1% Triton-X, 10% Normal Donkey Serum (NDS, Millipore S30, Temecula, CA, USA), 3% albumin bovine (BSA; Amresco Inc., Solon, OH, USA) in PBS. Primary antibody was added overnight in 4°C on shaker. Samples were then washed for 20 minutes six times in PBS at RT, and secondary antibodies were added for 2 hours on a shaker at RT. Samples were placed on slides with mounting medium after 4′,6′-diamidino-2-phénylindole (DAPI) (Enzo LifeScience Exeter, UK) staining, for cell nucleus identification. Flat mounts of eyes with LI-CNV and without primary antibodies served as negative controls, which defined our background for the microscopy.

**Figure 1:**
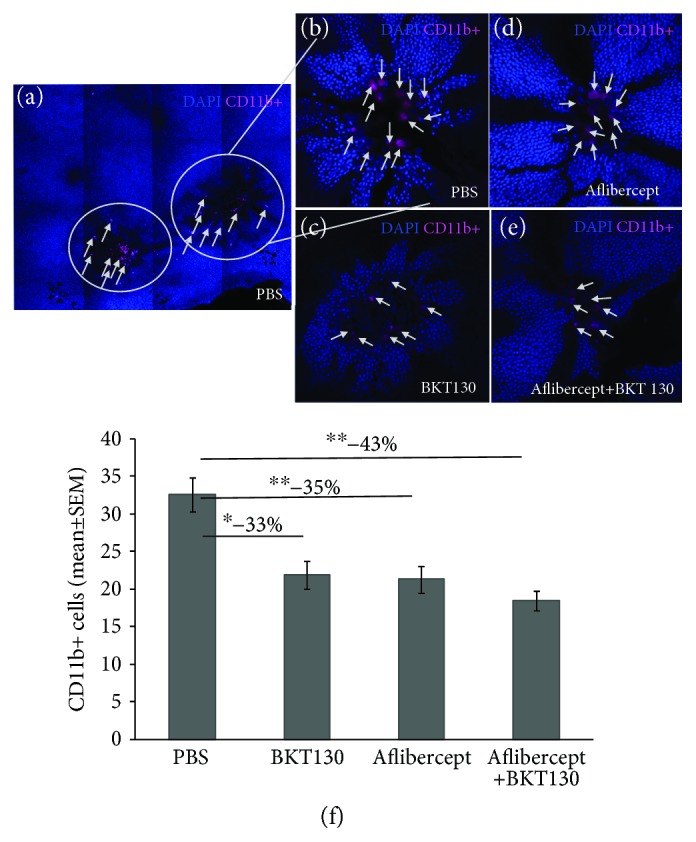
BKT130 reduces CD11b+ cell migration to the laser-treated area. Rat retinal flat mounts were prepared 10 days following laser injury and intravitreal injections. Flat mounts were observed using a confocal laser scanning system. CD11b-positive cells were observed in the center of the laser-treated areas (a). Each laser-treated area was observed in a 40x lens, and the macrophages (magenta) were counted (b–e). The eyes injected with BKT130, aflibercept, or BKT130+aflibercept demonstrated less CD11b-positive cells ((d) and (e), respectively) compared with PBS-injected eyes (b). A comparison between the amounts of cells found in laser-treated areas in each group is provided in (f). The *Y*-axis presents the mean (±SEM) number of CD11b+ cells found in each laser-treated area, in either BKT130 (number of laser burns = 19), aflibercept (*n* = 25 laser injury areas), BKT130+aflibercept (*n* = 41), or control PBS-injected group (*n* = 24). ^∗^ *P* < 0.05 and ^∗∗^ *P* < 0.005.

Immunofluorescence analysis was performed using a Zeiss LSM 710 confocal laser scanning system (Carl Zeiss MicroImaging GmbH, Jena, Germany) with 25X oil objective and a tile scan. Background was controlled by setting the exposure parameters as described above. These same parameters were maintained while capturing images from the test sections. CD11b+ cells which were found in the laser injury site, at the sub retinal space, were counted by a masked observer, using ImageJ software. The perimeter of the laser injury site was determined based on the absence of nearby photoreceptor cells as identified via DAPI staining, surrounding the laser injury (Figure 1(a)). Results are presented as the mean number of cells per laser-treated area of each experimental group ± SEM.

### 2.4. Quantitative Real-Time PCR (QPCR)

Total RNA was extracted from the flash-frozen retinas using TRI Reagent (Sigma-Aldrich), according to the manufacturer's instructions, and treated with DNase (TURBO DNA-free, Ambion, Austin, TX). Reverse transcriptase polymerase chain reaction was performed using the High Capacity cDNA Reverse Transcription Kits (Applied Biosystems, Foster City, CA) and anchored oligo dT primers on 1 *μ*g total RNA in a volume of 20 *μ*l.

Quantitative real-time PCR (QPCR) was performed using the SYBR Green technique to measure mRNA levels of genes involved in angiogenesis, inflammation, mononuclear cell marker, macrophage polarization, and monocyte recruitment. Oligonucleotide primers for genes of interest [*CCL2*, *CCR2*, *CCL5*, *VEGFA*, *IL1β*, *TNFα*, *NAP-2*, *MIP-2*, *CD11b*, *CD163*, *MRC1* (*CD206*), and *N0S1*] and for an endogenous control gene (*β-actin*) were designed for QPCR using Primer-Blast (https://www.ncbi.nlm.nih.gov/tools/primer-blast/). These genes were selected as they are related with chemokine signaling or with proangiogenic function of macrophages. All primers were purchased from Sigma-Aldrich (primer sequences are presented in Supplement Table 1). Measurement of the mRNA levels was performed on the retinas and RPE-choroid tissues, separately, 10 days after injections, in each experimental group (*n* = 9 eye samples in each group: PBS, BKT130, aflibercept, and, BKT130+aflibercept). Measurement of *β-actin* mRNA levels served as endogenous controls. All reactions were carried out in triplicate, using 384-well plates, at a total volume of 10 *μ*l. Wells contained 20 ng (for *CCL2*, *CCR2*, *CCL5*, *MRC1*, *CD163*, *VEGFA*, *CD11b*, *NOS1*, and *IL1β*) or 100 ng (for *TNFα*, *NAP-2*, and *MIP-2*) cDNA template, 5 *μ*l of SYBR Green FastMix (Quanta Biosciences), and 0.5 *μ*l forward and reverse primers (10 mM) for each gene. Signal amplification was measured throughout 38 cycles of 60°C for 20 seconds, followed by 95°C for 20 seconds. To confirm the amplification of a specific cDNA, the dissociation temperature was examined and compared with the calculated melting temperature for each amplified product. The amplified products were also examined by agarose gel electrophoresis. Fluorescent signals were measured by the CFX384, C1000 touch thermal cycler (Bio-Rad) and analyzed using the spreadsheet software (Excel; Microsoft, Redmond, WA). Expression levels of each gene were compared across the samples by using the expression levels of the endogenous control according to the standard 2^(-*ΔΔ*CT)^ calculation [57], giving results as relative quantification and fold change ± standard error of the mean (SEM).

### 2.5. Choroid Sprouting Assay (CSA)

Blood samples (20 ml) were collected from 6 nvAMD patients (3 females, 3 males, mean age ± SEM: 70.8 ± 2.3 years, range: 64-81) in EDTA tubes (BD Bioscience). Patients were recruited from the retina clinic of the Department of Ophthalmology at the Hadassah-Hebrew University Medical Center. The criteria for the inclusion of nvAMD patients included the following: age over 55 years, diagnosis of AMD according to the AREDS criteria [58], and diagnosis of CNV according to fluorescein angiogram and optical coherence tomography. All patients signed an informed consent form, and the study was approved by the institutional ethics committee (see Ethical Approval). Monocytes were isolated from the whole blood, differentiated into macrophages (M0), and activated into M1- and M2a-like phenotypes, as we and others have previously described [22, 59, 60].

An ex vivo angiogenesis assay was performed as previously described [22, 61], to evaluate the effect of BKT130 on the macrophages' proangiogenic phenotype. Briefly, the supernatant from human-activated and human-polarized macrophages that were treated with 50 *μ*g/ml BKT130 or untreated control macrophages was collected at day 7 of monocyte cell culture and kept in -20°C until use. Treatment with BKT130 took place at the day of macrophage polarization or at day 5 for the nonactivated macrophages (M0).

Adult C57BL6J mice, which were treated in accordance with the guidelines of the Association for Research in Vision and Ophthalmology (ARVO), were utilized for CSA experiments. Experiments were conducted with the approval of the institutional animal care ethics committee (see Ethical Approval). Mice were anesthetized with ketamine, checked for responses, and euthanized by cervical dislocation. The eyes were immediately enucleated and kept in ice-cold ECGS medium containing 1/100 penicillin-streptomycin and 1/100 glutamine before dissection. A choroid-sclera complex from the mice was gently dissected along with retinal pigment epithelium (RPE). The complex was cut into 5-6 1 mm long pieces. Fragments were embedded in 30 *μ*l of growth factor-reduced Matrigel™ (BD Biosciences, Cat. 354230) in 24-well plates. The thickness of the Matrigel™ was approximately 0.4 mm. Plates were then incubated for 10 minutes in 37°C, in a 5% CO_2_ cell culture incubator without medium to solidify the Matrigel™. Medium (250 *μ*l) containing ECGM (C-22010, PromoCell, Germany), 2.5% supplement mix (C-9215, PromoCell, Germany), 5% FCS, 1/100 penicillin-streptomycin, and 1/100 glutamine was added to each well. 250 *μ*l of the macrophages' supernatant or 250 *μ*l of medium only was added to each well in duplicates. In addition, BKT130 was added directly to another group of CSA wells with the supernatant of untreated polarized M0 and M1 macrophages from 5 other nvAMD patients (4 female, 1 male, mean age ± SEM: 77.8 ± 3.9 years, range: 64-87), to assess the effect of BKT130 directly on the supernatant without its potential effect on the macrophages' phenotype. Medium was changed every 3 days, and the cultures were fixed with 4% PFA after 8 days. Cultures were viewed with an inverted phase-contrast CKX41 Olympus microscope, and images were photographed with an Olympus DP70 digital camera (Olympus, Tokyo, Japan).

ImageJ software was used to quantify the sprouting area. The scale was set to convert pixels to mm^2^. Each image was converted to an 8-bit format to obtain a binary image. Sprouting area quantification and analysis were performed in duplicates for each sample.

### 2.6. Statistical Analysis

Data was processed using the biostatistical package InStat (GraphPad, San Diego, CA). *P* < 0.05 was considered to indicate the statistical significance. Values over two standard deviations from the average were excluded from the statistical analysis. Appropriate statistical tests were applied according to the results of a normalcy test, sample distribution, and nature of the parameters.

## 3. Results

### 3.1. BKT130 Suppresses Laser-Induced Model of CNV (LI-CNV)

**Figure 2:**
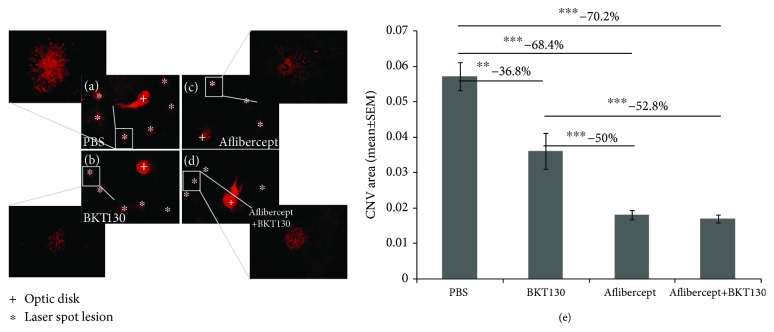
*In vivo* assessment of BKT130's effect in a rodent model of LI-CNV. BKT130 was injected intravitreally in a rat model of LI-CNV (*n* = 9 eyes). The eyes injected with PBS served as a negative control (*n* = 10), while intravitreal injections of aflibercept served as the positive control (*n* = 8). BKT130 was also injected with aflibercept to assess an additive effect (*n* = 8). CNV was identified and quantified using a fluorescent microscope in isolectin-stained RPE-choroid flat mounts (a–d). Each laser treated area was observed in a 20x lens and the whole flat mount in 4x lens. The CNV area was measured and compared between treatments and between PBS-injected eyes (e). The *Y*-axis presents the averaged (±SEM) CNV area (mm^2^) of treated and PBS-injected control eyes. ^∗∗^ *P* < 0.005 and ^∗∗∗^ *P* < 0.0005.

The LI-CNV rat was utilized to evaluate the *in vivo* effect of BKT130 on CNV growth (Figure 2). The CNV area was measured 10 days after the induction of LI-CNV and commencement of intravitreal therapy in the rat eyes. BKT130 treatment (*n* = 9 eyes) was associated with a 36.8% reduction in the CNV area [mean area (mm^2^) ± SEM] as compared with control (*n* = 10) PBS-injected eyes (0.036 ± 0.005 vs. 0.057 ± 0.004, respectively; *P* = 0.005, ANOVA). Aflibercept treatment (*n* = 8) was associated with a 68.4% reduction of the CNV area as compared with controls (0.018 ± 0.001, *P* = 0.0001). Injection of both aflibercept and BKT130 (*n* = 8) resulted in a 70.2% smaller CNV area (0.017 ± 0.001, *P* = 0.0001). CNV was 50% smaller in aflibercept-treated eyes compared with BKT130-treated eyes (*P* = 0.0003) and 52.8% smaller in aflibercept+BKT130-treated eyes (*P* = 0.0003).

### 3.2. BKT130 Inhibits Mononuclear Phagocyte Recruitment to a LI-CNV

Immunostaining for CD11b+ cells was performed on the photoreceptor side of the retina flat mounts to assess their recruitment to the LI-CNV (Figure 1). CD11b+ cells were found beneath the photoreceptors (between retinal and RPE cells) overlying the laser injury site (Figure 1(a)). Laser injury sites were of similar size across the experimental groups, while the number of CD11b+ cells was associated with the specific treatment provided (Figure 1(a)). The lowest CD11b+ cell count (43% reduction) was found in the aflibercept+BKT130 (number of laser-treated areas = 41; mean cell count in each laser-treated area ± SEM: 18.51 ± 1.26) as compared with the control PBS-treated eyes (*n* = 24 laser injury areas, mean cell count = 32.63 ± 2.23; *P* < 0.001; ANOVA). A reduction in the number of CD11b+ cells was also found in BKT130-treated eyes (*n* = 19, cell count = 21.89 ± 1.85; *P* < 0.01; ANOVA) and in aflibercept-treated eyes (*n* = 25, 21.36 ± 1.78; *P* < 0.001; ANOVA) as compared to PBS-treated eyes. No difference in the mean cell number was found among BKT130, aflibercept, and combined aflibercept+BKT130-treated eyes.

### 3.3. BKT130 Treatment Affects Gene Expression Profile in the Eyes with LI-CNV

**Figure 3:**
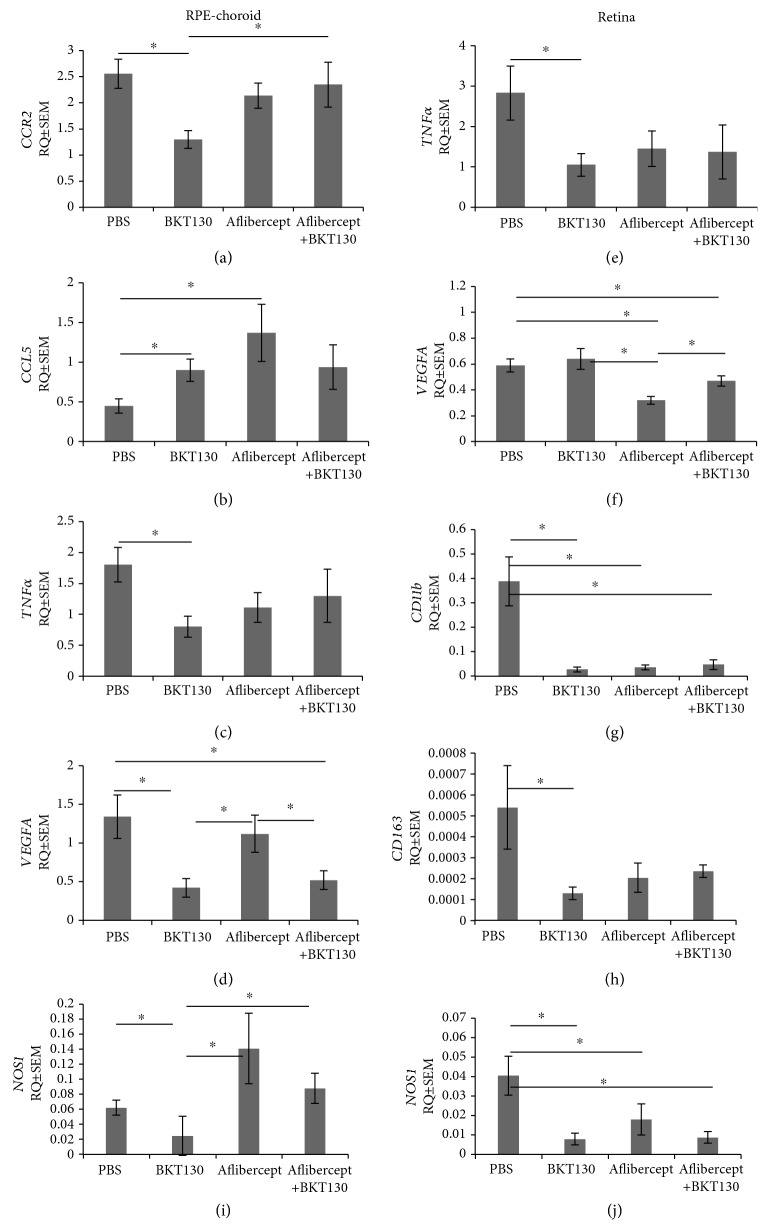
Gene expression profile of the retinas and RPE-choroid following treatment. mRNA expression levels of genes related to angiogenesis (*VEGFA*, *IL1β*, and *TNFα*), inflammation (*CCL2*, *CCR2*, *CCL5*, *TNFα*, *NAP-2*, and *MIP-2*), mononuclear cell markers (*NOS1*, *CD163*, and *CD11b*), and macrophage recruitment (*CCL2*, *CCR2*, *NAP-2*, and *MIP-2*) were evaluated in the RPE-choroid (a–d) and in the retinas (e–h) of rats via QPCR (*n* = 9 eyes in each group: PBS, BKT130, aflibercept, and BKT130+aflibercept). Presented are the genes that significantly changed after treatment. The *Y*-axis indicates RQ ± SEM. ^∗^ *P* < 0.05.

The mRNA expression profile of several genes evaluated with QPCR was associated with the specific treatments provided, as well as the tissue tested (retina and RPE-choroid; Figure 3).

Mean RPE-choroid *CCR2* expression (RQ ± SEM) was 2-fold lower in the BKT130-treated eyes (*n* = 9 eyes, 1.3 ± 0.26) as compared with PBS (*n* = 9, 2.56 ± 0.37, *P* = 0.02, *t*-test) and 1.8-fold lower from aflibercept+BKT130-treated eyes (*n* = 9, 2.35 ± 0.37, *P* = 0.05, *t*-test) (Figure 3(a)). Multivariate analysis for CCR2 mRNA levels in the RPE-choroid across the four groups did not disclose a difference (*P* = 0.2, Kruskal-Wallis test).

*CCL5* mRNA levels in the RPE-choroid was 2-fold higher in BKT130-treated eyes (0.9 ± 0.14, *P* = 0.03, *t*-test) and 3-fold higher in aflibercept-treated eyes (*n* = 9, 1.37 ± 0.36, *P* = 0.04, *t*-test), as compared to the eyes injected with PBS (0.45 ± 0.09; Figure 3(b)). Multivariate analysis for *CCL5* mRNA levels across the four groups in the RPE-choroid did not disclose a difference (*P* = 0.15, Kruskal-Wallis test).

*CCR2* and *CCL5* expression in the retinal tissue was similar among the experimental groups.

*TNFα* expression was 2.25-fold lower in RPE-choroid (0.8 ± 0.17) and 2.7-fold lower in retinal (1.05 ± 0.28) tissues of BKT130-treated eyes as compared to the PBS-treated eyes (1.8 ± 0.28, *P* = 0.03, Mann–Whitney test; 2.83 ± 0.67, *P* = 0.05, *t*-test, respectively) (Figures 3(c)–3(e)). A multivariate test for retinal *TNFα* expression across the four groups confirmed variable expression levels among the groups (*P* = 0.04, Kruskal-Wallis test). A multivariate test for RPE-choroid *TNFα* expression across the four groups did not disclose a difference (*P* = 0.1, Kruskal-Wallis test), yet when we pooled the two groups that were treated with BKT130 (BKT130 and aflibercept+BKT130), the multivariate test for RPE-choroid *TNFα* expression across the three groups confirmed variable expression levels among the groups (*P* = 0.04, Kruskal-Wallis test).

Retinal *VEGFA* expression was 1.8-fold lower in aflibercept-treated eyes (0.32 ± 0.03) as compared to PBS-treated eyes (0.59 ± 0.05, *P* = 0.0003, *t*-test). BKT130 treatment was associated with 3.2-fold reduced expression of *VEGFA* in the RPE-choroid (0.42 ± 0.12) as compared to PBS-treated eyes (1.34 ± 0.28, *P* = 0.02, *t*-test). The combination of aflibercept+BKT130 was associated with lower *VEGF*A expression by 1.25-fold in retinal tissue (0.47 ± 0.04) and 2.6-fold reduced levels in RPE-choroid tissue (0.52 ± 0.12) as compared with the PBS-treated group (0.59 ± 0.05, *P* = 0.05, *t*-test; 1.34 ± 0.28, *P* = 0.01, *t*-test, respectively) (Figures 3(d)–3(f)). A multivariate test for *VEGFA* expression across the four groups disclosed variable expression levels (*P* = 0.03 in the retina and *P* = 0.004 in the RPE-choroid, Kruskal-Wallis test).

A multivariate test for *CD11b* expression in the retina across the four groups disclosed variable expression levels (*P* = 0.003, Kruskal-Wallis test). Univariate analysis suggested that *CD11b* expression in the retina was decreased by 14-fold following BKT130 (0.026 ± 0.01) treatment and by 10-fold following aflibercept treatment (0.03 ± 0.01) as compared with PBS- (0.4 ± 0.1) treated eyes (*P* = 0.02, *t*-test; *P* = 0.02, *t*-test, respectively) (Figure 3(g)). No change was measured in *CD11b* expression in the RPE-choroid tissue.

A multivariate test for *CD163* expression in the retina across the four groups did not disclose a difference (*P* = 0.1, Kruskal-Wallis test). Univariate analysis demonstrated that BKT130 treatment was associated with a 4.17-fold reduction of *CD163* (an M2 macrophage biomarker) expression in the retina (0.0005 ± 0.0002 and 0.0001 ± 0.00003, *P* = 0.03, *t*-test, respectively) (Figure 3(h)). No change was measured in *CD163* expression in the RPE-choroid tissue.

In univariate analysis, BKT130 treatment was associated with reduced *NOS1* (an M1 macrophage biomarker) expression in the RPE-choroid and in the retina, respectively (RPE-choroid: BKT130—0.02 ± 0.02, PBS—0.06 ± 0.01, *P* = 0.05; retina: BKT130—0.008 ± 0.003, PBS—0.04 ± 0.01, *P* = 0.01, *t*-test). Aflibercept monotherapy was not associated with altered *NOS1* expression in the choroid or the retina tissues. The combination therapy of aflibercept+BKT130 was associated with reduced NOS1 levels in the retina (aflibercept+BKT130: 0.008 ± 0.003, PBS: 0.04 ± 0.01, *P* = 0.02, *t*-test), but not in the RPE-choroid. A multivariate test for *NOS1* expression in the retina and in the RPE-choroid across the four groups disclosed variable expression levels (*P* = 0.04 for both tissues, Kruskal-Wallis test) (Figures 3(i) and 3(j)).

No difference in the expression levels of *MRC1*, *CCL2*, *IL1β*, *NAP-2*, and *MIP-2* in the retina or in the RPE-choroid was identified across the treatment groups (data not shown).

### 3.4. BKT130 Does Not Affect Macrophages' Proangiogenic Phenotype or Function

**Figure 4:**
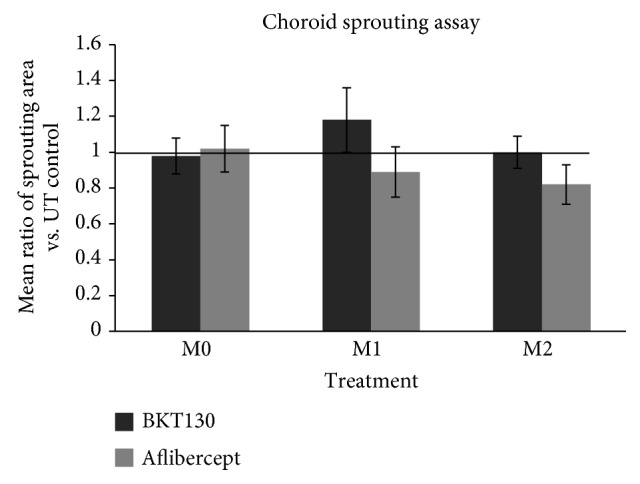
Choroid sprouting assay.

An ex vivo CSA was conducted to evaluate the effect of BKT130 on macrophage's proangiogenic phenotype and the function of the secreted proteins. No difference in the sprouting area was detected among wells treated with the supernatant of macrophages that were incubated with or without BKT130 (*n* = 6 in each group, mean of CSA area in mm^2^ ± SEM, M0: untreated 1.72 mm^2^ ± 0.32 vs. treated 1.64 mm^2^ ± 0.32, *P* = 0.7; M1: untreated 2.2 mm^2^ ± 0.34 vs. treated 2.2 ± 0.35, *P* = 0.4; M2: untreated 1.62 mm^2^ ± 0.34 vs. treated 1.54 mm^2^ ± 0.25, *P* = 0.6; paired *t*-test). In addition, the sprouting area was not affected by the addition of BKT130 to the CSA wells treated with macrophage's culture media in each macrophage subtype tested (mean of CSA area in mm^2^ ± SEM, M0: without BKT130 1.4 mm^2^ ± 0.6 vs. with BKT130 1.12 mm^2^ ± 0.6, *P* = 0.8; M1: without BKT130 2.2 mm^2^ ± 0.9 vs. with BKT130 1.36 mm^2^ ± 0.5, *P* = 0.5, paired *t*-test) (Figure 4).

## 4. Discussion

We describe the effect of a novel promiscuous chemokine antagonist (BKT130) in the rat model of LI-CNV. Application of this compound via the intravitreal route was associated with a reduction in the recruitment of CD11b+ cells to the proximity of CNV lesions, a reduction of CNV size, and suppression of the expression of chemokines and cytokines, including the major monocyte receptor—CCR2—in the RPE-choroid tissue. Despite the fact that BKT130 inhibits chemokines which are expressed not only by the inflamed tissue but also by M1 and M2 macrophages, ex vivo treatment with BKT130 in CSA or treatment of cultured macrophages with BKT130 failed to suppress choroidal sprouting.

These data suggest that BKT130's favorable *in vivo* effect is mediated via perturbation of chemokine signaling and monocyte recruitment to the laser-injured area. Recently, it was suggested that microglia are resident macrophages of the retina that are derived from embryonic yolk sac progenitors during development, while nonresident bone marrow-derived macrophages may be recruited into the retina from the vasculature in pathology [62]. Therefore, any additional CD11b+ cells found in the retina are likely to represent recruited macrophages rather than resident microglia [63]. Macrophages were implicated in the pathogenesis of AMD based on multiple studies, among them are the presence of macrophages in the vicinity of AMD lesions [44, 45, 64, 65], proangiogenic human and rodent macrophages' effect in vitro and in the rodent model of LI-CNV [18, 22, 66], and the reduced size of experimental CNV following inhibition of the CCR-CCL2 signaling pathway and monocyte recruitment [30, 67].

BKT130 suppresses LI-CNV via antagonizing multiple chemokines, thereby indirectly suppressing the expression of VEGF and other proinflammatory and proangiogenic cytokines. In the present study, anti-VEGF therapy was also associated with reduced macrophage recruitment, conceivably, through a PGF trap which inhibits subretinal phagocyte accumulation and other different mechanisms [68–70].

Macrophages may mediate CNV progression via cytokine production. TNF*α*-expressing macrophages were previously detected in CNVs excised from AMD patients [71]. Our previous study showed that M1 macrophages from nvAMD, which had a proangiogenic effect in the rat model of LI-CNV, also produce considerable amounts of TNF*α* [22]. In addition, it has been previously suggested that macrophages secreting TNF*α* in CNV stimulate RPE expression of *VEGF* [71, 72] and that TNF*α* increases the secretion of VEGF A and C and leads to the upregulation of *VEGF* expression by human RPE cells and choroidal fibroblasts [73, 74]. Our current results showed approximately 60% reduction in *TNFα* expression in both retinal and RPE-choroidal tissues following BKT130 treatment. In addition, we found reduced expression of *CD11b* and *NOS1* in both retinal and RPE-choroid tissues following BKT130 treatment which suggest reduced mononuclear phagocyte recruitment and reduced polarization towards the M1 macrophage phenotype. Interestingly, aflibercept, while suppressing macrophage recruitment as evident by reduced *CD11b* expression in the retina, did not suppress *TNFα, NOS1*, or *CCR2* expression suggesting that it did not affect macrophage polarization or polarized macrophages' recruitment. Thus, our findings support a potential suppression of M1 macrophages' polarization by BKT130 while aflibercept may potentially suppress the recruitment mononuclear cells but not polarization to the M1 phenotype and therefore may have a different mechanism of action on CNV growth. These results are in accordance with a recent report of higher expression of M1 markers in the RPE-choroid of a mouse following laser-induced CNV as compared to M2 macrophages' markers which were increased in the retina [75]. M1 macrophage activation and M1-dominant polarization profile of microglia were also recently described in the degenerative retina of rd1 mice [76].

VEGF immunoreactivity was previously found to be greater in inflammatory and active CNV and was found in the RPE to a greater extent than found in macrophages [77]. In addition to the RPE [78] and macrophages, at least six more retinal cell types have the capacity to produce and secrete VEGF including astrocytes [79], Müller cells [80], endothelial cells [81], microglia [77], pericytes [82], and ganglion cells [83]. BKT130 downregulated *VEGFA* expression in the RPE-choroid, but not in the retina. By contrast, aflibercept downregulated *VEGFA* expression in the retina and not in the RPE-choroid. Interestingly, combining aflibercept with BKT130 treatments caused downregulation of *VEGFA* expression both in retina and in RPE-choroid tissues. These results may reflect the variable mechanism of VEGF suppression associated with the two compounds and suggest a potential complementary effect of the combined therapy.

Caveats of the current study include the fact that LI-CNV in rat is a wound-healing reaction that follows an insult at the level of Bruch's membrane and relies heavily on inflammation [18, 19] and that it does not directly reflect nvAMD. In addition, because of the absence of a defined macula in rodents, this rodent model does not fully mimic the complexity of human pathology [84]. However, this model was proven to be suitable for testing the efficacy of new drugs through systemic or intraocular administration and has shown a predictive value for drug effects in patients with AMD, for example, with aflibercept [85, 86]. In addition, while we observed a trend towards enhanced suppression of CNV in the combination arm of aflibercept+BKT130, this arm did not show a smaller CNV size as compared with aflibercept monotherapy. Yet in the LI-CNV model, application of aflibercept essentially eliminated the neovascular tufts, thereby, resulting in a ceiling effect that does not allow for a functional effect of the combined therapy to be apparent. Such complete elimination of the CNV lesion is not usually achieved in nvAMD following anti-VEGF therapy. Thus, in the human pathology, there is a need for supplementing the effect of available therapies. Finally, the lower injection volume used in the aflibercept monotherapy group as compared to other groups may theoretically interact with CNV size. Yet our control group was injected with 4 *μ*l of PBS, similar to the BKT130 group which was the main focus of this research. Furthermore, the highest injection volume was used in the 1 *μ*l + 4 *μ*l of the BKT130+aflibercept group, and this group yielded suppression of CNV.

## 5. Conclusion

Intravitreal delivery of a promiscuous chemokine antagonist, BKT130, inhibited the recruitment of monocytes to the laser injury area, reduced CNV area in the LI-CNV rat model, and decreased expression of *VEGFA* and *CCR2* in RPE-choroid and *TNFα* in both RPE-choroid and retinal tissues. Reduction in *TNFα* and *NOS1* with BKT130 but not with aflibercept might suggest a different macrophage subtype inhibition and therefore an additional effect on different patients. Additionally, a combination therapy with BKT130 and anti-VEGF had an additive effect on *VEGFA* expression in the eyes of rats with LI-CNV. Future studies should evaluate if perturbation of chemokine signaling may serve as a novel therapeutic option in nvAMD to supplement anti-VEGF therapy.

## Data Availability

We are happy to provide any relevant raw data, to any scientist who wishes to use them. For more information, please contact the corresponding author at chowers@hadassah.org.il.

## Abbreviations

AMD: Age-related macular degeneration
nvAMD: Neovascular stage of AMD
RPE: Retinal pigment epithelium
CNV: Choroidal neovascularization
LI-CNV: Laser-induced model of choroidal neovascularization
CSA: Choroid sprouting assay
FCS: Fetal calf serum
VEGF: Vascular endothelial growth factor.

## Supplementary Materials

Supplementary Materials Appendix Table 1: mRNA primers for QPCR.
