# Supplementary material for: Promiscuous Chemokine Antagonist (BKT130) Suppresses Laser-Induced Choroidal Neovascularization by Inhibition of Monocyte Recruitment
Source: J Immunol Res. 2019 Aug 5;2019:8535273. doi: 10.1155/2019/8535273 (PMC6701410; doi:10.1155/2019/8535273)
Supplement: Supplementary Materials — Appendix Table 1: mRNA primers for QPCR. [file 8535273.f1.docx]

1. **Appendices Table 1: mRNA primers for QPCR**

| **Gene name** | **Gene synonym** | **Forward** | **Revers** |
| --- | --- | --- | --- |
| VEGF |  | TGGACCCTGGCTTTACTGCT | AATTGGACGGCAATAGCTGC |
| TNFα |  | GATCGGTCCCAACAAGGAGG | GCTTGGTGGTTTGCTACGAC |
| CCR2 |  | CCACACCCTGTTTCGCTGTA | CTGCATGGCCTGGTCTAAGT |
| IL1β |  | AGGCTGACAGACCCCAAAAG | CTCCACGGGCAAGACATAGG |
| MIP-2 | CXCL2 | ATCCAGAGCTTGACGGTGAC | TCCAGGTCAGTTAGCCTTGC |
| CCL5 | RANTES | TATGGCTCGGACACCACTCC | CACTTCTTCTCTGGGTTGGCA |
| NAP-2 | CXCL7 PPBP | TGCGCTGCAGATGTACGAAT | TTGATCATAGGGGCAGTCGG |
| CCL2 | MCP1 | TGATCCCAATGAGTCGGCTG | TGGACCCATTCCTTATTGGGG |
| CD163 |  | GACAGACCCAACGGCTTACA | TCTTAAATGCCAACCCGAGGA |
| CD206 | MRC1 | GAGGACTGCGTGGTGATGAA | GAGCGAACGTTGAAAGGGTG |
| NOS1 |  | AACGGGGAGAAATTCGGCTG | GGGAGAGGGAAGGCCCTTTA |
| β-actin |  | TGTGGCATCCATGAAACTAC | ATTTGCGGTGCACGATGGAG |
